# Restoring the Balance between Pro-Inflammatory and Anti-Inflammatory Cytokines in the Treatment of Rheumatoid Arthritis: New Insights from Animal Models

**DOI:** 10.3390/biomedicines10010044

**Published:** 2021-12-26

**Authors:** Adrienn Markovics, Ken S. Rosenthal, Katalin Mikecz, Roy E. Carambula, Jason C. Ciemielewski, Daniel H. Zimmerman

**Affiliations:** 1 Department of Orthopedic Surgery, Rush University Medical Center, Chicago, IL 60612, USA; Adrienn_Markovics@rush.edu (A.M.); Katalin_Mikecz@rush.edu (K.M.); 2 Department of Basic Sciences, Augusta University/University of Georgia Medical Partnership, Athens, GA 30602, USA; kenneth.rosenthal@uga.edu; 3 Department of Integrative Medical Sciences, NE Ohio Medical University, Rootstown, OH 44272, USA; 4 CEL-SCI Corporation, Vienna, VA 22182, USA; RCarambula@cel-sci.com (R.E.C.); JCiemielewski@cel-sci.com (J.C.C.)

**Keywords:** peptide vaccine, immunotherapy, inflammatory, anti-inflammatory, cytokines, rheumatoid arthritis, proteo-glycan (PG, aggrecan), PG G1 domain-induced arthritis, collagen-induced arthritis, animal models

## Abstract

Rheumatoid arthritis (RA) and other autoimmune inflammatory diseases are examples of imbalances within the immune system (disrupted homeostasis) that arise from the effects of an accumulation of environmental and habitual insults over a lifetime, combined with genetic predispositions. This review compares current immunotherapies—(1) disease-modifying anti-rheumatic drugs (DMARDs) and (2) Janus kinase (JAK) inhibitors (jakinibs)—to a newer approach—(3) therapeutic vaccines (using the LEAPS vaccine approach). The Ligand Epitope Antigen Presentation System (LEAPS) therapies are capable of inhibiting ongoing disease progression in animal models. Whereas DMARDs ablate or inhibit specific proinflammatory cytokines or cells and jakinibs inhibit the receptor activation cascade for expression of proinflammatory cytokines, the LEAPS therapeutic vaccines specifically modulate the ongoing antigen-specific, disease-driving, proinflammatory T memory cell responses. This decreases disease presentation and changes the cytokine conversation to decrease the expression of inflammatory cytokines (IL-17, IL-1(α or β), IL-6, IFN-γ, TNF-α) while increasing the expression of regulatory cytokines (IL-4, IL-10, TGF-β). This review refocuses the purpose of therapy for RA towards rebalancing the immune system rather than compromising specific components to stop disease. This review is intended to be thought provoking and look forward towards new therapeutic modalities rather than present a final definitive report.

## 1. From Homeostasis to Autoimmunity

Rheumatoid arthritis (RA) and other autoimmune inflammatory diseases are examples of imbalances within the immune system (disrupted homeostasis) that arise from the effects of an accumulation of environmental and habitual insults over a lifetime, combined with genetic predispositions. The initial insults that lead to RA are thought to often originate in the lung and are connected with smoking. Lungs are constantly exposed to insults due to infections, injury, and inhalants, dust, silica, asbestos, and especially tobacco smoke [1,2,3]. In addition, bacterial infections in the oropharynx, leading to gingivitis or periodontal disease, are considered a possible key early factor (see reviews) [1,4,5,6,7]. The inflammatory responses that drive RA usually originate with autoimmune responses against normal, modified or immuno-mimetics of self-proteins that are often found within skeletal joints. Individuals may be genetically prone to or acquire an enhanced systemic inflammatory state due to infectious, metabolic, or other challenges, which are often related to the immunological maintenance of the gut microbiome. The accumulation of inflammatory insults over a lifetime generates such a state, which has been termed ”inflammaging” [8].

## 2. Post-Translational Modification and Its Role in Autoimmunity 

Tobacco smoking and other challenges promote the activation of the enzyme peptidyl arginine deiminase (PADI, especially PADI-2 and PADI-4) in the lung and elsewhere. This enzyme converts arginine residues in proteins to citrulline in situ in a process called citrullination. Such post-translational modifications (PTM) can alter the immunogenicity of any protein containing that residue and create neo-epitopes, leading to the development of autoimmunity. Citrullinated vimentin, proteoglycan (PG), fibrin, and collagen are commonly found in the skeletal joints of RA patients [2]. Another PTM seen in RA involves lysine residues. Other in situ PTMs, such as on serine and threonine, alter the immunogenicity of other self-proteins and are being observed in a growing number of autoimmune diseases [1,2,3,9,10,11,12]. On an important note, PTM can affect cellular function and enhance the activation of neutrophils and macrophages, leading to the release of destructive enzymes from the recruited cells, such as matrix metalloproteinases (MMP, especially MMP-1, MMP-13), which are collagenases that can cause bone and cartilage destruction [13].

## 3. Role of Cytokines, Cells, and Their Interplay in Disrupting Immune Homeostasis

**Figure 1:**
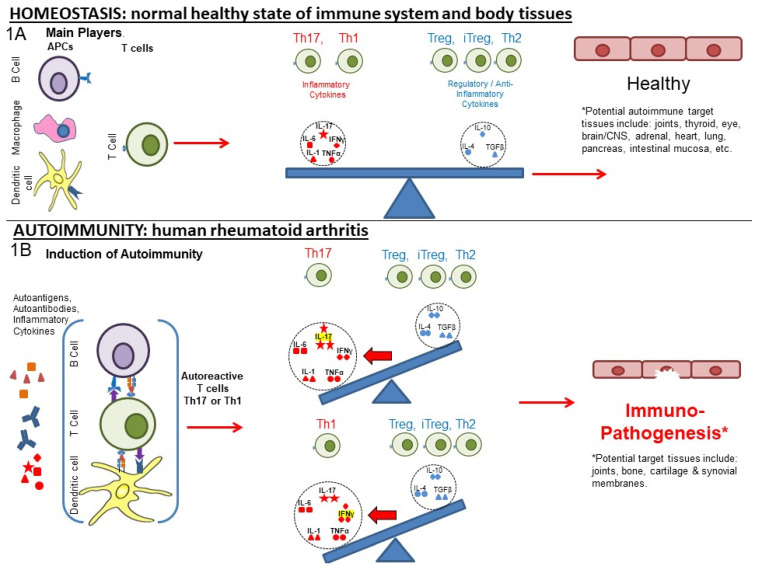
Disruption of the homeostasis of the immune system promoting arthritis. (1**A**) **Homeostasis:** Antigen-presenting cells present peptides and cytokines to activate antigen-specific T cells influenced by environmental signals and cytokines. A balance of pro-inflammatory to humoral and regulatory responses promote immune homeostasis. Red symbols within dotted circles represent pro-inflammatory cytokines and blue symbols represent anti-inflammatory cytokines. (1**B**) **Autoimmunity**: Environmental factors (e.g., smoking), trauma (repetitive bone or cartilage injury), infections (microbial antigen mimicry), and genetic predisposition (e.g., MHC: HLA-DR4) can promote an inflammatory environment that promotes a self-sustaining disruption of immune balance that can result in rheumatoid arthritis (RA). Arthritogenic self-antigens are presented by DCs, macrophages, and B cells to T cells to generate auto-antibodies and self-reactive T cells, respectively, which promote inflammatory cytokine production that activates other cells and induces tissue remodeling and disruption.

In the presence of systemic inflammation, potentially exacerbated by trauma, infection or other inflammatory trigger, PTM proteins can initiate autoimmune responses and alter the normal balance of immunity. Figure 1A represents the normal balance in immune pro-inflammatory and anti-inflammatory responses that promote immune homeostasis. The various actors include **cells** (T, and B cells, macrophages, dendritic cells (DC), other blood cells) and **pro-inflammatory and anti-inflammatory cytokines** that affect the regulation of responses to self (auto)antigen. 

As shown in Figure 1B, the disruption of immune homeostasis resulting in autoimmunity and RA can occur in an individual at risk of RA due to environmental, systemic, or genetic factors. The activation of inflammatory responses by exposure to the pathogen-associated molecular pattern molecules (PAMPs) of microbes or damage-associated molecular pattern molecules (DAMPs) induced by tissue damage or trauma can activate processes that can initiate inflammatory responses to the PTM self-(auto)antigens that drive RA. These processes are driven by T helper (Th)1 and/or Th17 pro-inflammatory responses. 

DCs process phagocytosed proteins, including citrullinated proteins if present, into peptides that then occupy major histocompatibility class (MHC I or MHC II) molecules and then activate the naive T cells that can recognize those peptide antigens. Depending on the cytokines produced by the DC, antigen-specific Th1 or Th17 pro-inflammatory T cells are activated [6,14]. These cell types are defined by different transcription factors (TFs) and the cytokines that they produce, setting up cytokine conversations. As summarized by Medzhitov, these conversations may include increases in some and decreases in other cytokines [15]. The cytokine conversation of Th17 T cells includes interleukins (IL), such as IL-17, IL-22 and tumor necrosis factor (TNF)-α, which can act on epithelial cells, neutrophils, and other cells, promoting epithelial cell functions, antimicrobial activities, wound healing or inflammation [1,16]. Activated neutrophils can cause and extend these inflammatory reactions. The cytokine conversation of Th1 cells includes interferon (IFN)-γ, IL-2, and TNF-β which has a broader role in activating CD4 and CD8 T cells, B cells, promoting immunoglobulin class switch to IgG and, importantly, the development of M1 inflammatory macrophages. M1 macrophages have increased capacity for producing reactive oxygen species, acute phase inflammatory cytokines (IL-1, TNF-α, IL-6) and they exhibit an enhanced antigen-presenting capacity [6,7,17]. Activated B cells produce antibodies to the RA autoantigens and are also potent activators of antigen-specific T cells in RA. B cells express the same immunoglobulin on their cell surfaces that they produce and secrete as the B cell receptor (BCR). This molecule, the BCR, is important for activating B cells and also promotes the phagocytosis of its corresponding antigen. The internalized antigen is then processed, and its peptides become the dominant peptide on MHC II molecules that are presented to and activate CD4 T cells, which can ultimately amplify the immune response to the antigen [5,18].

Normally, the B cells and T cells that elicit autoimmune responses are either eliminated in the bone marrow and thymus, respectively (central tolerance), or controlled by regulatory responses (peripheral tolerance). Peripheral tolerance is mediated by regulatory T cells (Tregs) and induced T regs (iTregs), whose cytokine conversations involve IL-10 and/or tumor growth factor (TGF)-β. The iTregs are generated primarily from peripheral Th17 or other Th cells in the presence of high levels of IL-10 or TGF-β. Other cells, including macrophages and myeloid suppressor cells, can also produce these regulatory cytokines to control inappropriate or inflammatory responses. The tolerance that these cells impose can be overridden by infections and other challenges that disrupt the homeostatic balance and lead to the production of large amounts of acute phase cytokines (IL-1, IL-6, TNF-α), allowing responses to the microbial or modified antigens that mimic human antigens to initiate autoimmune responses [5,19,20].

## 4. Two Different Animal Models of Arthritis

**Figure 2:**
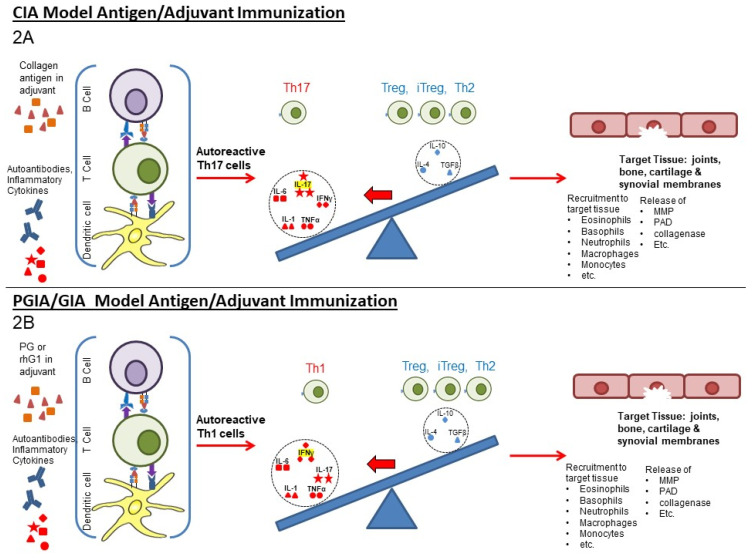
Overview of the CIA- and PGIA/GIA-induced animal models of rheumatoid arthritis (2**A**) **CIA:** The collagen-induced arthritis model is initiated by bovine collagen with adjuvant to promote inflammatory responses that override tolerance and induce autoimmune responses to collagen. The nature of the murine immune response and method of initiation induce a Th17-driven arthritis. (2**B**) **PGIA/GIA**: The proteoglycan-induced and the related rhG1 domain-induced models of arthritis are initiated in older female mice by injection of these proteins with adjuvant to promote Th1-driven arthritis which also includes Th17 responses. Human disease occurs mostly in older women and other aspects of this model also resemble human disease.

Animal models of RA, including collagen-induced arthritis (CIA), proteoglycan (PG) induced-arthritis (PGIA), and recombinant human PG G1 (rhG1) domain-induced arthritis (GIA) models, which are utilized in the studies described below, are useful for studying the disease and potential treatments. Autoimmune responses to RA-relevant antigens are initiated by immunization with collagen, PG, or immuno-dominant domains from these molecules in the presence of potent adjuvants that override tolerance. In the CIA model (Figure 2A), genetically susceptible male mice are immunized with type II bovine collagen emulsified in complete Freund’s adjuvant and then receive a boost injection 21 days later [21]. Arthritis develops 26–35 days after the initial collagen injection. In the PGIA model (Figure 2B), genetically susceptible (older female) mice are immunized with human cartilage PG in dimethyl-dioctadecyl-ammonium bromide (DDA) adjuvant three times with 21 day intervals, as described elsewhere [22,23]. In the related GIA model, mice are immunized with the recombinant first globular (G1) domain of human PG, also with the same adjuvant. The methods for disease induction (route, adjuvants, etc., used) are important in determining whether the disease has a Th1 (IFN-γ-dominated) or Th17 (IL-17-dominated plus TNF-α) phenotype with the involvement of the corresponding signature cytokines [24,25,26,27]. Characteristically, the CIA model is driven by Th17 pro-inflammatory cytokine conversations, while the PGIA/GIA model, used in the subsequently described studies, is driven by Th1 pro-inflammatory cytokine conversations. Similarly, it is likely that human joint disease, such as RA, is driven by either a Th1 or Th17 cytokine conversation. Many of the genetic predispositions seen in humans are also present in these and other animal models; see the review by Kurko et al. [28].

The CIA (Th17 with IL-17) and PGIA/GIA (Th1 with IFN-γ) mouse models of arthritis were used to explore the potential therapeutic effects of CEL-2000 (J-C-II_254_) and CEL-4000 (DerG-PG_70_) Ligand Epitope Antigen Presentation System (LEAPS) vaccines, respectively [3,27,29,30,31]. CEL-2000 [27] attaches a peptide from human collagen to the J-immune cell-binding ligand (ICBL) (J-C-II_254_) and CEL-4000 attaches a peptide from proteoglycan to the DerG-ICBL (DerG-PG_70_) [29,30].

The LEAPS vaccine therapy was developed as an alternative approach for the systemic ablation or inhibition of the immune responses involved in RA. Treatment with LEAPS promotes an antigen-specific modulation of cytokine conversations that reduces inflammation. LEAPS vaccines are peptides that can be designed to elicit an antigen-directed Th1 or Th2/Treg cytokine conversation depending upon the LEAPS ICBL that is attached to a disease-related antigenic peptide. The J-ICBL activates DCs, which produce IL-12 to promote IFN-γ and Th1 cytokine conversations and responses, whereas the DerG-ICBL acts on CD4 T cells to promote Th2 and Treg cytokine conversations and responses. By promoting the appropriate antigen-specific cytokine conversations and T cell responses, immunization with J-LEAPS vaccines can elicit anti-viral and anti-tumor responses [32,33] and also have the potential to modulate Th17 inflammatory responses [27]. The induction of IFN-γ and Th1 CD4 T cells activates anti-viral and anti-tumor cytotoxic T cells but can also downregulate Th17 responses. The DerG-LEAPS vaccines elicit antibody responses [34,35] and have the potential to modulate Th1 responses.

**Figure 3:**
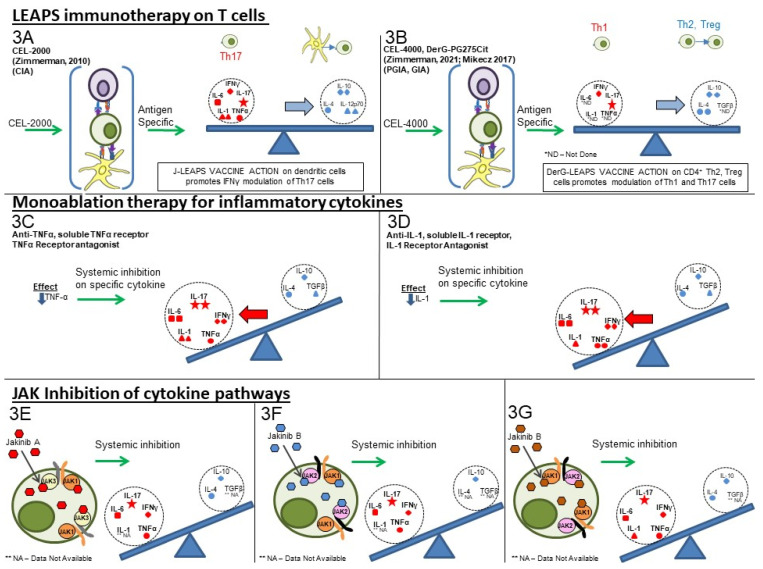
Comparison of cytokine-targeting therapies to treat autoimmune conditions. **LEAPS immunomodulating therapy:** (3**A**) ***CEL-2000 J-LEAPS vaccine:*** Immunization of diseased animals activates dendritic cells to promote antigen-specific Th1 responses and IL-10 to modulate the disease driving Th17 and inflammatory cytokine responses and provide therapy. (3**B**) ***CEL-4000 and related DerG-LEAPS vaccines**:* CEL-4000 vaccination of diseased animals activates antigen-specific CD4 Th2 and Treg cells to modulate the disease driving Th1, Th17 and inflammatory cytokine responses. Treatment favors a ratio of increased anti-(IL-4, IL-10) vs. pro-(IFN-γ or IL-17) for cytokine secreting CD4 spleen T cells. **Monoablation therapy for inflammatory cytokines (DMARDS):** (3**C**) ***Neutralizing antibody to* IL-1, TNFα, or IL-6 (not shown);** (3**D**) ***Receptor antagonist inhibition of cytokine action:*** Neutralization or blocking of cytokine receptor by antibody can prevent systemic action of a specific inflammatory cytokine but also affects antimicrobial and other immune responses. Treatment has no effect on anti-inflammatory cytokines. **Inhibition of JAK-tyrosine kinase cascade:** (3**E**–3**G**) ***Inhibitors of of different JAKs:*** Small molecular inhibitors of JAK1, JAK2, JAK3 or tyrosine kinase 2 (TYK2) block the signal transmission from associated cytokine receptors to block inflammatory and regulatory responses, depending upon the JAK(s) that are inhibited. These inhibitors downregulate transcription of one or more cytokine gene, as listed in Table 1.

As shown in Figure 3A, the application of LEAPS immunotherapy to disease-bearing mice in the animal models of RA promotes a return to a balanced immune response by promoting an increase in anti-inflammatory cytokines and a decrease in pro-inflammatory cytokines [3,29,30].

While the cartoon only shows a limited number of pro-inflammatory and anti-inflammatory cytokines as examples, the cytokine conversations in RA include IL-1 (α or β), IL-6, IL-17A (or IL-17F), IL-23, TNF-α, and IFN-γ for inflammatory. IL-23 is not included in the figure but does promote memory Th17 proinflammatory responses. And IL-4, IL-10 and TGF-β for anti-inflammatory cytokines. IFN-γ is unique as it can exhibit both pro- and anti-inflammatory roles. IFN-γ can activate inflammatory macrophages and T cells but also inhibits neutrophils and Th17 responses [17,24,36,37,38]. The LEAPS vaccines are presented as a more recent example of developing therapeutic vaccines in these same models, which modulate both inflammatory and anti-inflammatory cytokines as part of their action [3]. By modifying the cytokine conversation, the LEAPS immunotherapy is a better approach to restore immune homeostasis than single-cytokine ablation. As Smolen et al. [2] indicate, the cytokines produced by T cells may have both pro-inflammatory and anti-inflammatory activities depending upon the other cytokines within the “conversation” and the cell and tissue that is affected. Other articles on the LEAPS technology can be found at the following references: [3,32,39,40].

## 5. Current and Older Therapeutic Approaches for RA

The current therapy for RA consists of either the treatment of symptomatology or the inhibition or ablation of the components of the inflammatory immune response (Table 1). Neither of these approaches address the imbalance of the antigen-specific immune response that is the root cause of the disease. Non-steroidal anti-inflammatory drugs (NSAIDs) are the first line of treatment used to alleviate pain and inflammation. Older ablative treatments with corticosteroid, methotrexate, and similar drugs can inhibit inflammation and are effective for many patients. The newer therapies, as shown in Table 1, are more selective in their inhibition of specific mediators of inflammation and can be organized into three main therapeutic approaches, as shown in Table 1 and Figure 3.

## 6. Grouping of the Therapeutic Approaches 

The three groups of therapies shown in Table 1 are presented with the immunological target of the treatment, the cytokine(s) that are affected and whether their amount is increased or decreased and examples of specific drugs for that type of treatment. The information in Table 1 is presented as a “heat map” to assist in revealing trends and patterns. Highlighted in red are the five pro-inflammatory cytokines (IL-1(α or β), IL-6, IL-17, IFN-γ, and TNF-α), in Blue are two anti-inflammatory cytokines (IL-4 and IL-10) and in gray are IL-2, since it seems to play a key regulatory role, and IL-12p70, which promotes IFN-γ responses, which can be pro-inflammatory or anti-inflammatory. 

The Group **I** therapies focus on the cytokine-secreting, antigen-specific T cells, i.e., therapeutic vaccines, but not individual cytokines per se. These treatments promote a modification of the cytokine conversation that affects both anti-inflammatory cytokines and pro-inflammatory cytokines, focusing upstream on the source of the response, the memory T cell. 

Group **II** therapies focus on ablations of individual cytokine action by neutralizing the cytokine or blocking its receptor. Neutralizing monoclonal antibodies or receptor antagonists prevent the action of individual acute phase cytokines, TNF-α, IL-1, IL-6 or mediators of Th17 responses, such as IL-23 or IL-17. Alternatively, antibodies to surface differentiation antigens (CDs) are used to eliminate or inhibit specific cells and their functions. Antibody to CD20 lessens the number of B cells to reduce these RA-promoting, antigen-presenting cells. These therapeutic products, monoclonal antibodies, soluble receptor antagonists, agonists, or modified soluble receptors are collectively referred to as Disease-Modifying Antirheumatic Drugs (DMARDs).

The first developed and therefore oldest of the group **(II**) cytokine-focused therapies are the biological response modifiers (BRM), such as neutralizing monoclonal antibodies, solubilized receptors (sR) and modified soluble receptors (msR) (e.g., for IL-2R: MR-IL-2). As monoclonal antibodies, they are highly specific for the antigenic epitopes present on the target molecules [83,84] and act extracellularly. However, it should be noted that some protein subunits are shared between several cytokines, such as IL-12p40 for both IL-12 and IL-23 cytokines, so that multiple cytokines and their consequences may be affected [85,86]. Similarly, monoclonal antibodies directed towards cell surface markers (e.g., CD20) (other than cytokine receptors), may affect several different types of cells expressing that marker [48,84]. The Group II agents are often administered by injection as an intravenous (IV) bolus or infusion over time because of their much larger sizes (e.g., about 150 kDa for monoclonal antibodies).

Group **III** therapies focus on JAK inhibitors (abbreviated to jakinhib-1, -2 or -3, etc.) which target the JAK/STAT transduction of receptor signals, which activates the transcription and production of one or more cytokines, including not only the pro-inflammatory cytokines, but also potentially therapeutic anti-inflammatory cytokines, notably IL-2, IL-4, IL-10, and TGF-β [64,66,68,82,86,87,88,89]. The heat map is especially useful since specific drugs may have more activity towards one JAK than another, e.g., JAK3>1 family (for tofacitinib and for peficitinib); JAK2,1>TYK2 family (for baricitinib and for ruxolitinib); and JAK 1>JAK2 family (for filgotinib and for upadacitinib). TGF-β was not considered in this analysis as it is not involved in the JAK/STAT pathway.

## 7. Application of Animal Models in the Evaluation of Potential or Actual Therapies

RA and many other autoimmune diseases are driven by T cell responses directed towards specific epitopes from different proteins and possibly mediated by different T cell cytokine conversations. This situation can be seen for the two models of RA depicted in Figure 2 in which the CIA model is driven by Th17 responses to collagen and the PGIA/GIA model is driven by Th1 responses to proteoglycan. As in humans, the nature of the response is determined by multiple factors including genetics (for the mouse, it would be strain and breed [28]), diet, nature of antigen administration and level of systemic inflammation (possibly mimicked by the adjuvant). Similarly, other autoimmune conditions and their respective models, including uveitis, multiple sclerosis (MS), and autoimmune Type 1 diabetes (T1D) [3,24,25,26,90], are initiated by antigen responses and driven by the Th17 and/or Th1 pro-inflammatory immune responses, some of which also involve the tissues of bone/cartilage of the joints, as seen in these two RA models. 

In as much as many of the therapies for RA and other autoimmune and inflammatory diseases target the cytokines involved in the process, it is important to have an overview of the consequences of their inhibition or ablation. The depictions in the figures simplify the disease-driving cytokine conversations and the collateral effects that they induce. Many of the individual cytokines have multiple activities depending upon the cell type and the other cytokines in the conversation, as was mentioned for IFN-γ, for which ablation in some animal models for RA, diabetes and multiple sclerosis actually exacerbated disease [36]. Similarly, several cytokine receptors or their subunits bind multiple cytokines and block the receptor subunit or its transduction cascade, or the cytokine itself can have diverse consequences. Additionally, when considering T cell responses, it is important to note that multiple cytokines of different types can be secreted by a single T or other cell type [91]. These cytokines may be produced in multiple locations which may be distant from the initial trigger and have different effects depending upon the target tissue. The actual damage may be inflicted in a joint, skin, eye, pancreas or nervous tissue [1,2,3]. Immunopathogenesis can present as erythema, increased temperature, vascular permeability, swelling, pain, itching and, importantly, the recruitment of innate immune cell types such as eosinophils, neutrophils, basophils, and monocytes [2]. A further note is that since cytokine actions can have multiple effects, there can be a bystander effect [92,93,94], be it good or harmful, on adjacent tissues, depending on whether they are pro-inflammatory or anti-inflammatory. Relevant to RA, pro-inflammatory cytokines can cause other cells to release enzymes such as PADI-2 and PADI-4 (which generate neo-antigens) or more destructive enzymes such as MMP-1, MMP-13, and other collagenases that break down proteins in the bone or cartilage tissue, leading to joint destruction. This effect creates and releases new antigens which some experts consider to be the process called “epitope spreading” [2,95,96,97].

## 8. Comparisons of LEAPS, Monoablative, and Jakinib Therapies

Unlike the ablative therapy alluded to above, the LEAPS peptide therapeutic vaccines have an immunomodulatory effect on the T cells driving the disease, as illustrated in Figure 3A,B. In so doing, the LEAPS peptides affect the entire cytokine conversation, increasing the expression of some and decreasing other cytokines to return immunobalance, rather than acting on a single cytokine [3,27,29,30,31].

The monoablative therapies (shown in Figure 3C,D) using a neutralizing monoclonal antibody or receptor-antagonist to specifically block the action of one of the disease-associated cytokines after secretion and not its synthesis. The targets for these therapies include IL-1β, IL-6, IL-17A, IL-23, IL-12, and TNF-α and, under certain circumstances, IFN-γ. These monoablation therapies only indirectly affect other pro-inflammatory cytokines and do not upregulate anti-inflammatory cytokines to rebalance the cytokine conversation. More recently, it has been reported that immunotherapies based on IL-6 [98] or IL-17 [16] may be more efficacious for RA [2,99] than the original TNF-α antagonists exemplified by Adalimumab (Humira®) [100,101], Etanercept (Enbrel®) [102,103], or Infliximab (Remicade®) [102,103,104,105,106].

Targeting these individual cytokines also suppresses beneficial responses, including antimicrobial responses, that can impact GI immunology and antimicrobial protections. For example, the inhibition of TNF-α, IFN-γ, IL-17, IL-23, or IL-12 increases the risk of intracellular bacterial and fungal infections [107,108,109].

The third group (**III**) of therapies (Table 1, Figure 3E–G) are the jakinibs, which act by inhibiting specific receptor-associated JAK/STAT tyrosine kinases, ultimately inhibiting the synthesis and secretion of multiple cytokines (multi-ablative therapy) that are activated by the specific JAK cascade. The jakinibs are small-molecule (~300Da) inhibitors acting on the Janus kinases JAK1, JAK2, JAK3 or TYK2 and have the major advantage of being taken orally [63]. The JAK enzymes most often work in pairs as homo- or heterocomplexes activating STAT molecules to create transcriptional activators and promote the expression of groups of cytokine and other genes. JAK activation or inhibition also influences the expression of different cell surface receptors, including CD4, CD80 and CD86 on T, B and other cells and their associated immune responses [65]. The expression of some JAK enzymes is more restricted to certain cell types than others, such as JAK3 for immune system cells such as B, T, and NK cells. This is a therapeutic advantage.

As can be seen in Table 1, there are at least five different jakinibs approved for RA treatment in the USA, Europe, or Japan and several others are under investigation, each unique in terms of the molecule’s binding preference for its particular cognate JAK or JAK-associated molecule. Different manifestations of treatment occur depending on the relative selectivity of binding and whether it is reversible or irreversible. The representative jakinhibs are shown as different-colored (red, blue and orange) hexagonal shapes in Figure 3 for the three examples of jakinibs that downregulate inflammatory and anti-inflammatory cytokines. Jakinib A (Figure 3E **red hexagon**) has activity which is JAK 3>1 and downregulates the expression of IL-6, IL-17, IFN-γ, TNF-α, IL-4, and IL-10. Jakinib B (Figure 3F **blue hexagon**) is a JAK 2>1 inhibitor downregulating IL-6, IL-17, IFN-γ, TNF-α, and IL-10. Jakinib C (Figure 3G **orange hexagon**) is a JAK 1>2 inhibitor downregulating IL-1, IL-6, IL-17, IFN-γ, TNF-α, IL-4, and IL-10. It should be noted that a jakinib specific for only JAK2 cannot be used since the inhibition of JAK2/STAT is associated with lethality early in life [63,77].

As discussed by Lin et al. [77], the specificity for the different targets of JAK classes (1,2,3 and TYK2) and the magnitude of inhibition of each can result in a spill-over of JAK inhibition, affecting multiple activities. This can result in the inhibition of expression of beneficial and anti-inflammatory cytokines, such as IL-10 (controlled by the JAK1/TYK2 pair), IL-12p70 (controlled by the JAK2/TYK2 pair), GM-CSF (controlled by the JAK2 pair), or IFN-γ (controlled by the JAK1/JAK2 pair) by a particular jakinib [64,66,68,69,82,86,88,89,110]. Likewise, the ablative effects of monoclonal antibodies and soluble cytokine receptor antagonists may extend beyond their action on the specific cytokine target to also affect cell proliferation and surface molecule expression [47,57].

Although TNF-α, IL-1, and IL-17 are major targets for monoablation therapy, they are not directly affected by the jakinibs. However, they may be indirectly affected by the inhibition of expression of other cytokines that are supposedly not involved in the JAK signaling pathway; for example, IL-17 is affected indirectly by several of these jakinibs [66,67,77]. Similarly, an indirect effect of jakinibs may also promote the expression of some anti-inflammatory cytokines. The combination therapy targeting several cytokines, as possibly seen for the JAK inhibitors, may be effective, although this is still being debated and new clinical studies will be needed [2,99,111,112]. 

## 9. More on LEAPS and Therapeutic Vaccines and Summary

The LEAPS therapy, the development of which started after the other treatments listed in Table 1, has the major advantages of being antigen specific and thus disease specific, and directed towards specific T cell responses to adjust the cytokine conversation. The treatment acts by upregulating the anti-inflammatory cytokine conversation while at same time modulating or downregulating the expression of pro-inflammatory or inflammatory cytokines. Pharmacologically, the LEAPS peptides are sized between the other two classes of therapeutics (3–4kDa) and are injected subcutaneously or intramuscularly, more like a vaccine instead of given as an intravenous bolus or infusion.

The choice of the LEAPS ICBL attached to the disease-related peptide allows the designing of a therapeutic that modulates different pro-inflammatory responses. As shown by Taylor et al., who used J-LEAPS conjugates of epitopes from herpes simplex virus (HSV) and human immunodeficiency virus (HIV), the J-LEAPS peptides bind to precursors of mouse (bone marrow) or human (blood monocytes) DCs and promote their maturation to produce IL-12p70 and present the attached peptide to elicit antigen-specific protective responses, including the production of IFN-γ [113]. This Th1 cytokine conversation and involvement of both CD4 and CD8 T cells allowed the designing of both anti-viral and anti-tumor vaccines [32,33]. Similarly, CEL-2000, which attaches a collagen peptide to the J-LEAPS ICBL, upregulated IL-12p70, IFN-γ, and also IL-10 while downregulating TNF-α, IL-6, and IL-17A to stop the progression of ongoing disease in the CIA model [27]. LEAPS therapeutics incorporating the DerG peptide act directly on CD4 T cells to promote Th2 cytokine responses. CEL-4000 and a related LEAPS therapeutic, which attach the DerG LEAPS ICBL to peptides from proteoglycan [3,29,30], elicited antigen-specific responses to stop the progression of disease by acting on memory T cells, including those in the spleen, to increase the expression of anti-inflammatory IL-4, IL-10, and TGF-β cytokines and Treg numbers while decreasing the expression of pro-inflammatory IFN-γ and IL-17 in the PGIA/GIA models [29,30].

Immunomodulation as a therapy for inflammatory diseases, as demonstrated by the LEAPS vaccines, is a much newer approach than the DMARD or jakinib approaches. LEAPS vaccines have proven to be successful in representative animal models and J-LEAPS peptides have demonstrated activity with human cells [113] but have not been tested in humans. The genetic heterogeneity of the human population compared to research animals and an appropriate dosing regimen may pose potential challenges for the translation of LEAPS vaccine therapy from animal models to humans. As proposed in the work of Rosenthal et al. [114], the answer may be a customized therapy designed after testing the cytokine response of peripheral blood from patients upon challenge with a panel of representative autoantigenic peptides in a manner similar to the IFN-γ release assay (IGRA) for tuberculosis. The assay would uncover the disease-driving T-cell response for individual patients (Th1 or Th17) and allow the selection of the corresponding LEAPS ICBL and target antigen to incorporate into a customized therapy. For a Th1-driven disease, the LEAPS-4000 approach using the DerG-LEAPS ICBL would be appropriate, whereas, for a Th17, a LEAPS-2000 approach using the J-LEAPS ICBL would be appropriate.

The LEAPS approach derives benefits by addressing the underlying disease cause and not just being palliative or treating the symptoms of the disease. Rather than acting directly on pro-inflammatory cytokines, the LEAPS peptides act on DCs or T cells to modulate the ongoing disease-driving responses in an antigen-specific manner.

In conclusion, these examples of LEAPS immunomodulator peptides represent a new class of therapeutics for autoimmune inflammatory diseases that can be tailored to the disease-driving antigen(s) within an individual. This is in contrast to either of the ablation technologies that neutralize or block specific cytokines or block the JAK activation of gene expression for pro-inflammatory cytokines, with no consideration of affecting anti-inflammatory cytokines, and they do this in an antigen non-specific manner.

## Figures and Tables

**Table 1:** Approaches to targeting inflammatory cytokines in RA and RA animal models with regard to targets, cytokines, and therapies.

Type	Target	↓/↑ Modulation	Regulated Immune Component, If Known [References]	Generic and Product name, Regulatory Status	Ref.
Therapeutic Vaccines	Th1	↓	IL-1, IL-17, IFN-γ, TNF-α [3,29,30]	CEL-4000 (preclinical)	[3,29,30]
		↑	Treg (FOXP3+), IL-4, IL-10, TGF-β [3,29,30]		
	Th17	↓	TNF-α, IL-17, IL-6, MCP-1, IL-12p40 [27]	CEL-2000 (preclinical)	[27]
		↑	IL-12p70, IL-10 [27]		
DMARDs	TNF-α	↓	TNF-α [41]	Adalimumab (Humira®)	[41,42]
	TNF-α	↓	TNF-α [43]	Etanercept (Enbrel®)	[43]
	IL-1Ra	↓	IL-1 [44]	Anakinra (Kineret®)	[44]
	IL-6R msR	↓	MCP-1 [42], IL-6 [45]	Tocilizumab (Actemra®)	[42,45]
	IL-17	↓	MCP-1 [42], IL-17A [46]	Secukinumab (Cosentyx®)	[42,46]
	CD20	↓	B cells as APCs: CD4+IFN-γ+, CD4+IL-17+ [47]	Rituximab (Rituxan®)	[47,48]
	Anti-CD6	↓	IL-17 [49], IFN-γ [49,50], IL-6, TNF-α [50]	Itolizumab (Alzumab®)	[49,50,51]
	Agonistic Anti-CD137	↑	IFN-γ [52,53], IDO [53]	Utomilumab	[52,53]
	Anti-CTLA4	↓	IL-17, IFN-γ [54]	Abatacept (Orencia®)	[54,55,56]
		↑	IL-35, IFN-β [54]		
	Anti-CD40	↓	IL-6, RANKL [57], TNF-α, NF-κβ, IL-6, ICAM-1, VCAM-1, VEGF [58]	Bi 655064	[57,58]
	CD24	↓	TNF-α, IL-6, MCP-1(CCL2), IL-1β [59] NF-κβ [60]		[59,61,62]
Jakinibs	JAK3 > JAK1, JAK2 > TYK2 [63]	↓	**Transcription**: IL-2, IL-4, IL-7, IL-9, IL-15, IL-21, IL-6, IL-11, IL-13, IL-25, IL-27, IL-31, IFN-α, IFN-β, IL-10, IL-22, IFN-γ, > EPO, TPO, GH, G-CSF, GM-CSF, Leptin, IL-3, IL-5 > IL-12, IL-23, Type III IFNs [64] **in vitro**: IL-6 by B cells, [65] IL-2, IL-4, IL-7, IL-15, IL-21, IL-6, and IFN-γ in CD4+ T cells. IL-17 in Th17 cells polarized via IL-23. IL-21 and IL-22 in Th17 [66], IFN-α, IL-6, IFN-γ, IL-2, IL-15, IL-4, GM-CSF [64] MCP-1 [42] IL-17 in CD4+T cells from AS, PSA, RA, and HC [67] **in vivo**: IL-6 in human [68]	Tofacitinib (Xeljanz®)FDA approved (2012)	[42,63,64,65,66,67,68,69,70,71]
		↑	**in vitro**: IL-2 in Th1. IL-17, IL-2 in Th17 cells (polarized via TGF-β1, IL-6) [66]		
	JAK3 > JAK1, TYK2, JAK2 [63]	↓	**Transcription**: IFN-α, IFN-β, IL-10, IL-22, IL-2, IL-4, IL-7, IL-9, IL-15, IL-2, IFN-γ > IL-6, IL-11, IL-13, IL-25, IL-27, IL-31, IL-12, IL-23, Type III IFNs, EPO, TPO, GH, G-CSF, GM-CSF, Leptin, IL-3, IL-5 **in vitro**: IL-4, IL-13, IFN-γ, TNF-α in PBMC after TCR stimulation, IL-4, IL-13, IFN-γ, TNF-α, IL-17A, GM-CSF in PBMC after IL-2 stimulation [72]	Peficitinib (Smyraf®) Japan Approved (2019)	[63,72]
	JAK2, JAK1 > TYK2 > JAK3 [63]	↓	**Transcription**: IL-6, IL-11, IL-13, IL-25, IL-27, IL-31, IFN-α, IFN-β, IL-10, IL-22, IFN-γ > IL-2, IL-4, IL-7, IL-9, IL-15, IL-21 > IL-12, IL-23, Type III IFNs, EPO, TPO, GH, G-CSF, GM-CSF, Leptin, IL-3, IL-5 [64] **in vitro**: IL-6 in MoDCs, IFN-α secreted pDCs [65] MCP-1 [42] IL-17 in CD4+ T cells (AS, PSA, RA, and HC) [67]	Baricitnib (Olumiant®) FDA approved (2018)	[42,63,64,65,67,69,70]
	JAK2, JAK1 > TYK2 > JAK3 [63]	↓	**Transcription**: IFN-γ, EPO, TPO, GH, G-CSF, GM-CSF, Leptin, IL-3, IL-5 > IL-6, IL-11, IL-13, IL-25, IL-27, IL-31, IFN-α, IFN-β, IL-10, IL-22, IL-12, IL-23, Type III IFNs > IL-2, IL-4, IL-7, IL-9, IL-15, IL-21 **in vitro**: IL-10, IFN-γ, IL-6, TNF-α, IL-13 [73] IL-17 in CD4+ (AS, PSA, RA, and HC) [67] **in vivo**: IFN-γ, IL-12p70, IL-6, G-CSF, IL-10, TNF-α [73]	Ruxolitinib (Jakafi®) FDA approved (2011) (myelofibrosis)	[63,67,69,73,74]
		↑	**in vitro**: IL-2 [74]		
	JAK1 > JAK2 > TYK2 > JAK3 [63]	↓	**Transcription**: IL-6, IL-11, IL-13, IL-25, IL-27, IL-31 > IFN-α, IFN-β, IL-10, IL-22 > IFN-γ, > IL-2, IL-4, IL-7, IL-9, IL-15, IL-21 > EPO, TPO, GH, G-CSF, GM-CSF, Leptin, IL-3, IL-5, IL-12, IL-23, Type III IFNs [64] **in vitro**: IL-2, IL-4, IFN-αB2, IFN-γ [71] IFN-α, IL-6, IFN-γ, IL-2, IL-15, IL-4 [64] **ex vivo**: IL-6, GM-CSF [64] **in vivo**: IFN-γ, IL-6, IL-1β, RANKL, MMP-3, MMP-13, IP10, XCL1, MCP-1, MIP-1b, MCP-3, MCP-5, M-CSF1, MDC, SCF, KC/GRO, IL-1α [71] SAA, IL-6, IL-1β, GM-CSF, TNF-RI, Resistin, TNF-α, MMP-3, YKL40, VEGF, MMP-1, IL-12, IL-2, IFN-γ, IL-13, IL-5, IL-21, IL-23, IL-17A, IL-7, IL-10, CXCL10, CXCL13, MCP-1, VCAM-1, MIP-1a [75]	Filgotinib (Jyseleca®) EMA & Japan approved (2020)	[63,64,69,71,75]
	JAK1 > JAK2 > JAK3 > TYK2 [63]	↓	**Transcription**: IL-6, IL-11, IL-13, IL-25, IL-27, IL-31, IFN-α, IFN-β, IL-10, IL-22, IFN-γ EPO, TPO, GH, G-CSF, GM-CSF, Leptin, IL-3, IL-5 IL-2, IL-4, IL-7, IL-9, IL-15, IL-21 > IL-12, IL-23, Type III IFNs [64] **in vitro**: IFN-α, IL-6, IFN-γ, IL-2, IL-4, IL-15, G-CSF [64]	Upadacitinib (Rinvoq®) FDA approved (2019)	[63,64,70]
	JAK2 > JAK1 > TYK2 > JAK3 [76]	↓	**See main text** **in vitro**: VCAM-1, IL-6 [74]	Fedratinib (Inrebic®) (2019) (myelofibrosis)	[63,74,77,78,79]
		↑	**in vitro**: IL-2 [74]		

Footnotes: Heat map colors: Red: proinflammatory; Blue: anti-inflammatory; Gray: either possibility. For abbreviations used above, see abbreviation section before the author contribution section *For jakinibs:* extrapolated expectations are based on the inhibited JAK/STAT pathways indicated by experimental data. **Canonical JAK signaling pathways**: *JAK1/JAK3*: IL-2, IL-4, IL-7, IL-9, IL-15, IL-21; *JAK1/JAK2*: IFN-γ; *JAK1/TYK2*: IFN-α, IFN-β, IL-10, IL-22; *JAK1/JAK2/TYK2*: IL-6, IL-11, IL-13, IL-25, IL-27, IL-31; *JAK2/TYK2*: IL-12, IL-23, Type III IFNs; *JAK2/JAK2*: EPO, TPO, GH, G-CSF, GM-CSF, Leptin, IL-3, IL-5 [68,80,81,82].

## Data Availability

Not applicable.

## Abbreviations

AS: ankylosing spondylitis; BCR: B cell receptor; BRM: biological response modifier; CD: cluster of differentiation; CIA: collagen-induced arthritis; DAMP: damage associated molecular pattern; DC: dendritic cells; DDA: dimethyl-dioctadecyl-ammonium; DMARDs: disease-modifying anti-rheumatic drugs; EPO: erythropoietin; G1: globular 1; G-CSF: granulocyte colony-stimulating factor; GIA: PG G1 domain-induced arthritis; GM-CSF: granulocyte–macrophage colony-stimulating factor; HC: healthy controls; HIV: human immunodeficiency virus; HSV: herpes simplex virus; ICBL: immune cell-binding ligand; IDO: indoleamine 2,3-dioxygenase; IFN: interferon; IGRA: IFN-γ release assay; IL: interleukin; IP-10/CXCL10: interferon-inducible protein 10/C-X-C motif chemokine ligand 10; iTreg: induced regulatory T cell; IV: intravenous; JAK: janus kinase; KC/GRO: keratinocyte chemoattractant grow-regulated oncogenes; LEAPS: Ligand Epitope Antigen Presentation System; mAb: monoclonal antibody; MCP: monocyte chemoattractant protein; MDC: macrophage-derived chemokine; MHC: major histocompatibility class; MI: macrophage inflammatory protein; MMP: matrix metalloproteinases; MoDC: monocyte-derived dendritic cell; MS: multiple sclerosis; msR: modified soluble receptor; NSAID: non-steroidal anti-inflammatory drug; PADI: peptidyl arginine deaminase; PAMP: pathogen-associated molecular pattern; pcJIA: polyarticular course juvenile idiopathic arthritis; pDC: plasmacytoid dendritic cell;, PG: proteo-glycan; PGIA: proteoglycan-induced arthritis; PSA: psoriatic arthritis; PTM: post-translational modifications; RA: rheumatoid arthritis; RANKL: receptor activator of nuclear factor kappa-B ligand; rhG1: recombinant human PG G1; SCF: stem cell factor; sR: solubilized receptor; sRA: solubilized receptor antagonist; STAT: signal transducer and activator of transcription; T1D: type 1 diabetes; TF: transcription factors; TGF: tumor growth factor; Th: T helper; TNF: tumor necrosis factor; TPO: thrombopoietin; TRAP: tartrate-resistant acid phosphatase; Treg: regulatory T cell; TYK2: tyrosine kinase 2; UC: ulcerative colitis; XCL1: X-C motif chemokine ligand 1.
